# Bone Mass and Bone Quality Are Altered by Hypoactivity in the Chicken

**DOI:** 10.1371/journal.pone.0116763

**Published:** 2015-01-30

**Authors:** Eric Aguado, Florence Pascaretti-Grizon, Eric Goyenvalle, Maurice Audran, Daniel Chappard

**Affiliations:** 1 ONIRIS, Ecole Nationale Vétérinaire, Unité « Biologie et Biomatériaux du Tissu Osseux – Chirurgie Expérimentale ». route de Gachet, 44307 Nantes Cedex 3 – France; 2 GEROM (Groupe Etudes Remodelage Osseux et bioMatériaux), LHEA (Laboratoire d’Histologie Embryologie) IBS (Institut de Biologie en Santé), CHU d’Angers, LUNAM Université, 49933 Angers Cedex—France; Delft University of Technology (TUDelft), NETHERLANDS

## Abstract

Disuse induces a rapid bone loss in adults; sedentarity is now recognized as a risk factor for osteoporosis. Hypoactivity or confinement also decrease bone mass in adults but their effects are largely unknown and only few animal models have been described. We have used 10 chickens of the rapidly growing strain 857K bred in a large enclosure (FREE group); 10 others were confined in small cages with little space to move around (HYPO group). They were sacrificed at 53 days and femurs and tibias were evaluated by texture analysis, dual energy X-ray densitometry, microcomputed tomography (microCT) and histomorphometry. Hypoactivity had no effect on the length and diameter of the bones. Bone mineral density (BMD), microCT (trabecular bone volume and trabecular microarchitecture) and texture analysis were always found significantly reduced in the animals of the HYPO group. BMD was reduced at both femur and tibia diaphysises; BMD of the metaphysis was significantly reduced in the femur but not in the tibia. An increase in osteoid volume and surfaces was noted in the HYPO group. However, there was no alteration of the mineral phase as the osteoid thickness did not differ from control animals. Bone loss was much more pronounced at the lower femur metaphysis than at the upper metaphysis of the tibia. At the tibia, only microarchitectural changes of trabecular bone could be evidenced. The confined chicken represents a new method for the study of hypodynamia since these animals do not have surgical lesions.

## Introduction

The skeleton performs a variety of functions (mechanical, Ca/P reservoir, host tissue for bone marrow, endocrine organ…); the relative importance of which will change depending on environmental circumstances. Disuse, as observed after tetraplegia, paraplegia, denervation or poliomyelitis, is known to induce a rapid bone loss in adults [1]. It reduces bone mass and can create macro-anatomic changes in the growing skeleton [2]. Hypoactivity has also been found to decrease bone mass in adults [3] and sedentarity is recognized as a risk factor for osteoporosis [4]. Experimental immobilisation in healthy subjects (such as a long term bed-rest) has confirmed that unloading has deleterious effects on the musculoskeletal system [5]. In human, amputation of a leg segment induces a bone loss at the homolateral pelvis but neurovascular changes are considerable in the moignon [6].

Animal models are very helpful to better understand the pathophysiology of bone loss in various clinical conditions. Several models are based on surgical methods. Sectioning the motor nerve supply to a muscle produces a state of inactivity and atrophy in some muscles while others undergo a compensatory hypertrophy. Other surgical methods include tendon section, complete spinal cord section or hemi-cordotomy and have been reviewed elsewhere [7]. A muscle immobilization (by leg bandaging or casting) is also known to affect the muscle mass with subsequent bone loss [8]. However these animal models are often associated with neurological and sympathetic changes. The hindlimb suspension model has been proposed to mimic the effects of microgravity in bone that develops counter-measures [9, 10, 11, 12]. But, this model is rather difficult to develop and nowadays appears borderline in term of ethical consideration by animal-care committees. However, in all these models, disuse creates a combined association of a depressed osteoblastic activity and a sudden rise in bone resorption [7, 13, 14, 15, 16, 17]. We have proposed the BTX model in which a single intra-muscular injection of *Clostridium botulinum* toxin type A (BTX) provokes a severe unilateral amyotrophy leading to a rapid bone loss [18]. The model was also adapted in mice and rabbits by others [19, 20, 21]. The method is based on a reversible alterations of muscle fibers which can undergo recovery after the cessation of the BTX effects after several weeks. An increase in gene expression of resorption markers together with a decrease in gene expression of formation markers were shown in the mouse [22]. However, although the BTX model is atraumatic for the animals, functional and anatomical changes exist at the neuromuscular synapse with nerve de-afferentiation followed by muscle atrophy.

The aim of the present study was to analyze the effects of a prolonged hypoactivity, without nerve or muscle lesion, in a group of growing broiler chicken. The effects of disuse on bone mass and bone quality in these young growing animals have previously been rarely explored.

## Material and Methods

### Animals

The experiment protocol was approved by the Regional ethical committee at Oniris, National Veterinary School. Ten chickens of the rapidly growing strain 857K were grown in a large enclosure where they could walk freely (group FREE); 10 others were kept in a small cage with little space to move around (group HYPO). Both groups were housed in the same conditions of temperature (near 20°C) and light cycle (12h/12h light/dark cycle). All chickens were given equal amount of a normal poultry food with a normal calcium, phosphorus and vitamin D content. Chickens were sacrificed at 53 days of age by electronarcosis (the time to reach maturation and poultry marketing). Both femurs and both tibias were excised, fixed for 10 days in 10% buffered formalin and the mean value of all parameters was taken for statistical analysis.

### X-ray radiographs and texture analysis

The lower femur and the upper tibia extremities were radiographed on a Faxitron X-ray system (Edimex, Angers, France) with a 5 × 5 cm CCD camera which provided digitized images (1,024 × 1,024 pixels large) in the TIF format (Tagged-Image File format). The upper tibia epiphysis and the lower femoral epiphysis were analyzed. The accelerating voltage was fixed at 30 kV, 30 mA with a 9-second exposure time; the gain and offset were maintained at the same levels for the whole series of bones. The distance between the X-ray tube and the camera was constant and mechanically provided by the apparatus shelf with a magnification of × 5.

Texture analysis of X-ray images was used to appreciate bone microarchitecture with fractal algorithms developed in our laboratory [23, 24]. Texture analysis is a powerful tool to investigate bone microarchitecture at the 2D level on plain radiographs. The method was found interesting in BTX paralyzed rats and is more accurate than dual energy X-ray absorptiometry to evidence trabecular bone changes [18].

- The skyscraper algorithm is very useful to investigate the consequences of bone loss on the trabecular network. Briefly, a bitmap image can also be considered as a x*y surface made of pixels. It can be viewed as skyscrapers, imaged with an airplane point of view whose height is represented by the grey level [25]. The roof of the skyscrapers is a square of side ε = one pixel. The surface area of the image is obtained by measuring the sum of the top surfaces and sum of the exposed lateral sides of the skyscrapers. The grey levels of adjacent pixels are then averaged in squares of larger sizes (e.g., 4, 8, 16…) to produce new images and the new surface is calculated at each step. The fractal dimension (also known as the Kolmogorov’s fractal dimension) of the surface is determined by plotting a log-log graph of the surface areas against ε. The linear regression line was computed only on the aligned points by the least square method. The fractal dimension was obtained as D_SKY_ = 2—slope.
- The blanket method allows the study along the main direction of trabeculae by using dilatation and erosion of an image with 3 types of structuring elements gliding in the image: a cross, a horizontal and a vertical vector. Given a structuring element of size ε, a dilatation and an erosion of the image provides two new covering images: respectively, the upper and the lower ones. The volume of the blanket, i.e., the volume enclosed between the dilatation and erosion images, was measured. The number of dilations and erosions were ranged between 1 to 10 and the volume was measured each time. The fractal dimension D_blank_ was computed by plotting the logarithm of the volume against the logarithm of ε and searching the slope coefficient by the least-squares method. D_blank_ obtained with the cross (D+) and those obtained with a horizontal (D—) or vertical (D│) vector were determined.

The fractal dimension of an image is between 2 and 3.

### Dual energy X-ray absorptiometry (DXA)

Measurements were done on a Hologic QDR 4500A (Hologic Inc., Waltham, MA) with the small animal software (release V8-26h). Bones were placed in a vial filled with 5cm of water. BMD was assessed at the distal metaphysis of the femur and at the proximal tibia metaphysis using a proximal height resolution option of 0.0267 cm in line spacing with the small animal software. Cortical BMD was determined at the mid-shaft of each bone. The analysis was conducted on bones of the left and right sides and the mean value was taken for statistical analysis.

### Microcomputed X-ray tomography (microCT)

MicroCT was performed on the distal right femur with a Skyscan 1072 X-ray computed microtomograph (Bruker MicroCT, Kontich, Belgium) in the cone beam acquisition mode. Bones, still in the fixative to prevent desiccation, were scanned according to a routine protocol at 69kV, 100 µA and a 0.5mm aluminum filter. The pixel size was fixed at 19.28 µm and a 0.25° rotation angle was applied at each step. For each bone, a stack of 2D-sections was obtained. The CTAn Software (Skyscan, release 1.10.1.0) was used to measure the bone mass and architecture at the secondary spongiosa of the tibia. The first image selected for analysis was just under the growth plate (excluding the primary spongiosa), and then 668 2D sections were selected for the femur and 800 sections for the tibia. The volume of interest (VOI) was designed by interactively drawing a polygon on each 2D section. Only a few number of polygons need to be drawn (e.g. on the first section, several at the middle, and on the final section) since a routine facility calculated all the intermediary masks by interpolation. The VOI comprised only trabecular bone and marrow cavity. The following parameters were measured in the VOI (TV, in mm^3^) according to the recommendations of the American Society for Bone and Mineral Research [26].

- Trabecular bone volume (BV/TV_3D_, in %) represents the percentage of the cancellous space occupied by trabecular bone in the VOI.
- Microarchitectural parameters including trabecular thickness (Tb.Th, expressed in µm), trabecular number (Tb.N, expressed in 1/mm), trabecular separation (Tb.Sp, expressed in µm).

### Histological analysis

The left femurs were embedded undecalcified in poly (methylmethacrylate). They were cut-dry (7 µm in thickness) on a heavy-duty microtome equipped with tungsten carbide knife (Leica Polycut S-Rueil Malmaison, France). Sections were stained with a modified Goldner’s trichrome. All histological techniques have been extensively described elsewhere [27]. Histomorphometric analysis was done on a Leica Quantimet Q550 image processor at the secondary spongiosa where the bone trabeculae do not contain remnants of calcified cartilage. In order to appreciate the bone mass and spatial distribution of trabeculae, their connectivity and complexity the following measurements were calculated:

- Cortical thickness (C.Th, in µm),
- Trabecular bone volume (BV/TV_2D_; expressed in %),
- Strut analysis with determination of the total number of nodes (N), node-to-node branches (NN), node-to-free-end branches (NF). Parameters obtained were expressed as percent of the total strut length [28]. In order to obtain a single parameter for easy handling, the node to free-end ratio was determined (N/F) [29].
- Relative osteoid volume (OV/BV, expressed as %),
- Osteoid surface (OS/BS, expressed as %),
- Osteoid thickness (O.Th, expressed in µm).

### Statistical analysis

Statistical analysis was performed with Systat statistical software, release 13 (Systat Software, Inc., San José, CA). Data are expressed as mean ± SEM. Differences between groups were analyzed by the non-parametric Mann and Whitney U test. Differences were considered significant when p<0.05.

## Results

### Gross anatomy

No difference between left/right bone lengths and diameters was observed in both groups of animals. As such, the mean of both sides was used for inter-group comparison. No differences in length or diameters were noted between FREE and HYPO chickens at both the femur and tibia (Table 1).

**Table 1:** Gross anatomical measures of the tibia and femur in both groups of chicken.

**Parameters**	**Unit**	**FREE**	**HYPO**	**p**
Femur length	mm	83.0 ± 2.2	81.0 ± 3.6	NS
Tibia length	mm	113 ± 2.3	111.0 ± 4.2	NS
Femur diameter	mm	8.49 ± 0.39	8.82 ± 0.48	NS
Tibia diameter	mm	8.53 ± 0.48	8.48 ± 0.66	NS

### Texture analysis of X-ray films

**Fig 1:**
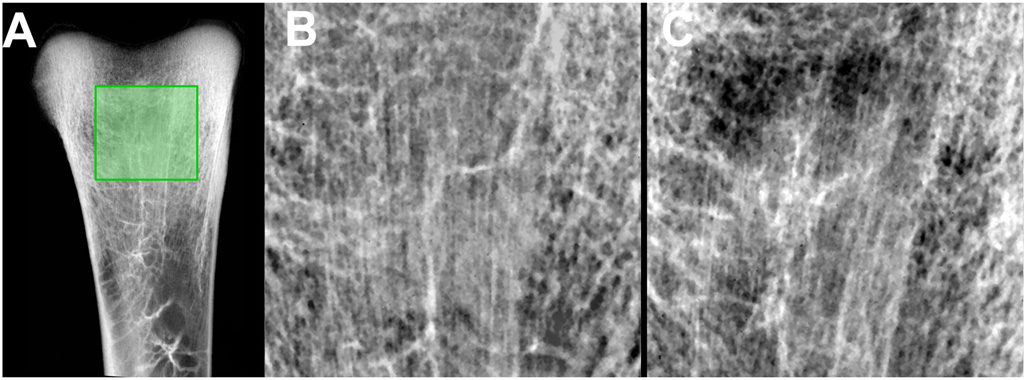
X-ray image of the region of interest at the tibia used for determination of fractal dimensions. A) General view of the upper bone extremity, the region of interest is delineated by the green frame. Note the complex distribution of the trabeculae with a vertical bundle of trabeculae extending into the cortical bone. B) Chichen of the FREE group, C) chicken of the HYPO group, note the marked alteration of the trabecular architecture.

Texture analysis performed on high resolution images clearly evidenced a loss of trabeculae in HYPO chickens (Fig. 1). However, the orientation of trabeculae appeared more complex than in mammals’ metaphysis with large vertical bundles of trabeculae extending into the diaphysis. Results, obtained by fractal analysis, evidenced altered bone microarchitecture in HYPO chickens with a reduction in the complexity of the trabecular network (Table 2). A disappearance of longitudinal and transverse trabeculae can be inferred from the reduction of the D_blank_— and D_blank_│parameters. No difference for D_sky_ could be evidenced in the HYPO group but the fractal dimensions obtained with the blanket method were all significantly different.

**Table 2:** Texture analysis of the femur and tibia X-rays.

**Parameters**	**FREE**	**HYPO**	**p**
Femur D_sky_	2.388 ± 0.003	2.376 ± 0.002	<0.001
Tibia D_sky_	2.359 ± 0.002	2.357 ± 0.002	NS
Tibia D_bank_+	2.423 ± 0.003	2.411 ± 0.002	<0.001
Femur D_bank_—	2.508 ± 0.005	2.471 ± 0.003	<0.001
Femur D_bank_+	2.455 ± 0.004	2.422 ± 0.002	<0.001
Tibia D_bank_│	2.445 ± 0.003	2.435 ± 0.002	<0.001
Femur D_bank_│	2.431 ± 0.003	2.409 ± 0.003	<0.001
Tibia D_bank_—	2.455 ± 0.003	2.443 ± 0.002	<0.001

### DXA measurements

**Fig 2:**
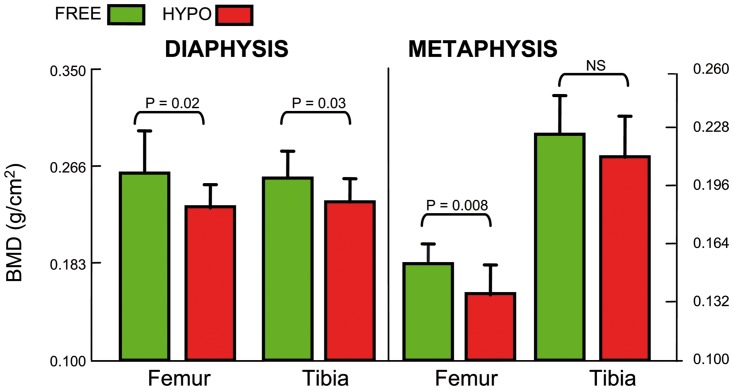
DXA analysis in the femur and tibia of FREE and HYPO chickens. Measurements were done at the mid-diaphysis for evaluation of cortical bone and at the lower femur metaphysis and upper tibia metaphysis for trabecular bone.

Because no differences in the left and right sides could be evidenced in each animal, the mean value was considered for inter-group comparison. A significant reduction in BMD was observed at the diaphysis in the HYPO group both at the femur and tibia (Fig. 2). At the metaphysis, the difference was significant in the femur but did not reached significance in the tibia. DXA analysis revealed a reduced BMD in hypodynamic animals that was significant both at the diaphysis (cortical bone) and at the metaphysis (trabecular bone). This reduction was more pronounced in the femur than in the tibia.

### MicroCT analysis and histomorphometric analysis

**Fig 3:**
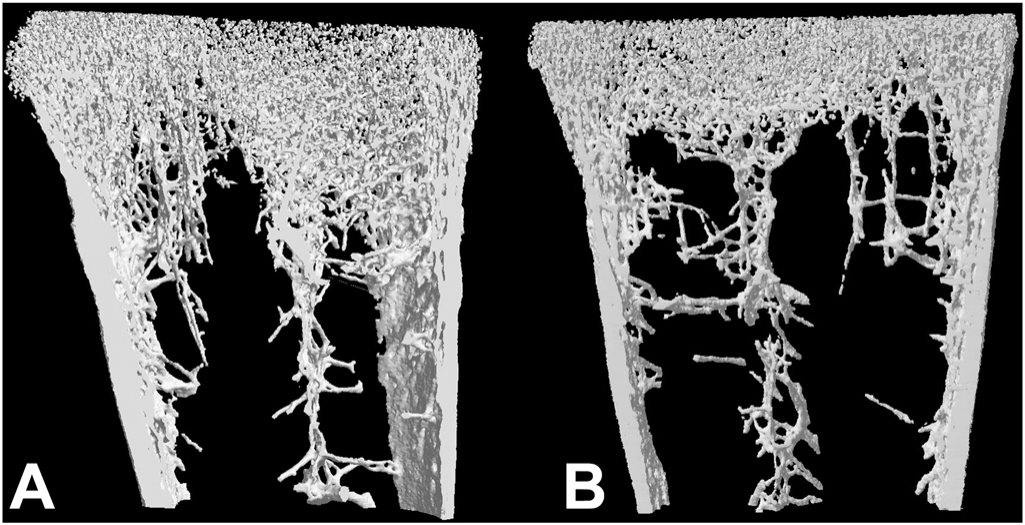
MicroCT analysis of the lower femoral metaphysis in A) chicken of the FREE group, B) chicken of the HYPO group. Note the marked reduction in the amount of trabecular bone in the HYPO group and the presence of a large bundle of vertical trabeculae extending to the diaphysis.

**Fig 4:**
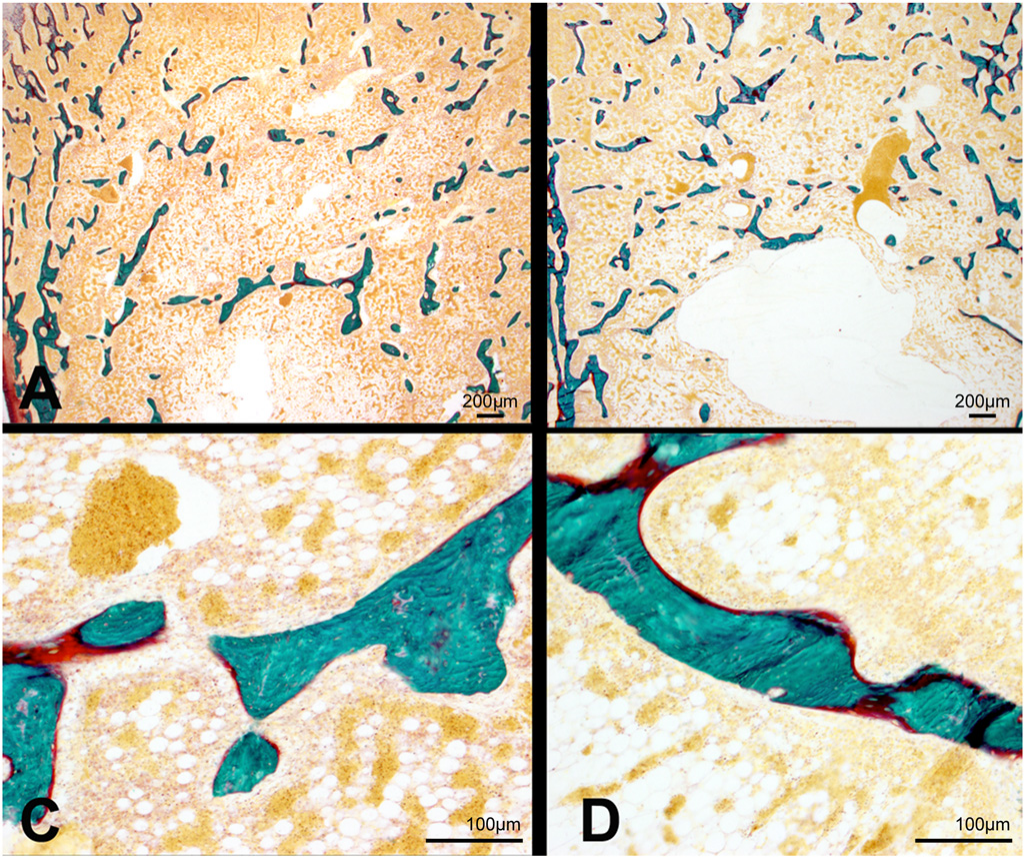
Histological aspect of trabecular bone at the metaphysis of A) and C) chicken of the FREE group and B and D) chicken of the HYPO group. Note the increase in osteoid volume and osteoid surfaces in the HYPO group. Goldner’s trichrome on undecalcified bone sections.

Histomorphometric results appear on table 3. Morphometric analysis was performed both in 2D and 3D since the techniques are known to provide complementary results [30]. A significant reduction in the trabecular bone volume could be evidenced both in 2D and 3D. Microarchitecture of the trabecular network appeared constituted of very thin trabeculae (Fig. 3) in the secondary spongiosa with a large bundle of trabeculae in the central part of the metaphysis extending into the diaphysis. Cortical thickness was not significantly reduced in the HYPO group on histological sections. Quantitative analysis evidenced a net reduction in the number of trabeculae with a concomitant increase in Tb.Sp in the HYPO group. However, no significant modification in trabecular thickness was observed. Microarchitectural deterioration was also confirmed by the strut analysis with a reduction in the node count, an increase in the Free-end struts and consequently a significant reduction in N/F ratio. Osteoid parameters were altered with a significant increase in OV/BV and OS/BS (Fig. 4). However, O.Th was similar in both groups, meaning that no mineralization defect was observed.

**Table 3:** histomorphometric results obtained on 2D histological sections or on 3D models prepared by microCT.

**Parameter**	**Unit**	**HYPO**	**FREE**	**p**
**2D femur**				
BV/TV	%	6.55 ± 1.43	8.07 ± 2.04	0.04
Ct.Th	µm	406 ± 45	424 ± 52	NS
Node-to-Free	-	0.40 ± 0.04	0.49 ± 0.08	0.02
OV/BV	%	10.0 ± 1.6	6.0 ± 0.9	0.001
OS/BS	%	36.4 ± 4.0	26.6 ± 6.0	0.007
O.Th	µm	5.4 ± 0.5	4.6 ± 0.8	NS
**3D femur**				
BV/TV	%	9.6 ± 1.6	10.6 ± 0.6	0.04
Tb.Th	µm	63 ± 4	60 ± 4	NS
Tb.N	/mm	6.1 ± 0.9	6.9 ± 1.1	0.04
Tb.Sp	µm	106 ± 14	89 ± 14	0.04
**3D tibia**				
BV/TV	%	5.4 ±1.03	4.2 ± 1.1	NS
Tb.Th	µm	63 ± 5	60 ± 3	NS
Tb.N	/mm	6.1 ± 0.8	6.8 ± 0.9	0.04
Tb.Sp	µm	106 ± 25	89 ± 17	0.04

## Discussion

A number of organs of the neuro-musculo-tendinous unit, when altered, can provoke a bone loss. Spinal cord, nerves, muscle tendons can be surgically sectionned and lead to the same consequences in bone. However, if these models may represent true clinical conditions, they always combine a surgical lesion and additional physiologic disturbances known to interact [31]. For example, paraplegia in human (or spinal cord section in animal model) is associated with muscle paralysis and local metabolic acidosis.

For these reasons, a considerable amount of work has been done in the search of animal models that could reproduce the effect of hypodynamia as observed in space flights or during sedentarity in human without muscle, nerve or tendon lesions. Simulated weightlessness also decreases bone mass in mature animals [9, 10] but has little effects on the bone growth in young animals [32]. This was also reproduced in avian models with simulated microgravity [33]. The BTX model was proposed as a non-surgical method and it induces histological alterations of synapses and muscles leading to a secondary massive bone loss [34, 35]. The present study describes an animal model without neurological, muscular, tendinous or bone alteration surgically-, pharmacologically- or mechanically-induced. In the present study, chickens bred in a restricted area exhibited a significant bone loss in the tibia and femur that could be evidenced by DXA, texture analysis, histomorphometry and microCT. The most striking finding was that bone loss interested both trabecular and cortical bone but neither the animal weight nor the gross anatomical bone measurements were altered at the end of the growth period. In poultry farming, some conditions such as an increased amount of proteins in the diet or a reduced lighting period can modify the macro-architecture of the long bones by inducing bone deformities [36]. This results in valgus or varus distortions of tibias and femurs with a “bow-legged” or “knock-kneed” appearance [37, 38]. In the present study, no such bone deformity was observed at the tibia and femur and no joint deformity could be observed on X-rays, microCT or after hand sectioning of the metaphysis. Trabecular bone loss was more pronounced at the femur as evidenced by all the techniques but it could be detected at the tibia only by microCT (reduction in Tb.N and increase in Tb.Sp) and texture analysis with the blanket algorithm. Texture analysis and microCT were performed at the lower femur and upper tibia epiphysis which are classical region-of-interest in rodent animal models of osteopenia [39]. These areas are also recognized as most valuable sites in bone research in avian bones [40] because they contain a high amount of trabecular bone which contributes to the mechanical strength of the long bones [41].

As previously reported, the fractal skyscraper algorithm is more suitable when trabeculae are isotropically disposed (with a “honey comb” appearance) which was the case in the femur metaphysis [18]. On the other hand, the structuring element used in the blanket algorithm is more adapted to the evaluation of trabeculae arranged anisotropically, as seen in the chicken tibia.

Another specific characteristic of the present model is that the effects of hypodynamia did not interfere with the growing skeleton of the animals. A very limited number of studies have been done on growing animals to evaluate the effects of hypodynamia caused by microgravity. In the BTX rat model, young rats did not have abnormalities of growth or alteration of the normal bone curvature [35]. In confined growing pigs, similar findings were observed without significant modification in body weight, tibia length but with a net reduction in cortical thickness due to a reduced periosteal apposition [42]. In children, temporary brittle bone disease may be due to an intra-uterine confinement with reduced fetal movements. This causes a decrease in bone density observed by radiographic absorptiometry or computed tomography in almost all cases [43]. In confined horses, radiographic absorptiometry also confirmed a significant decreased in bone density [44].

In this model, another alteration in bone quality was highlighted by a hyper-osteoidosis without mineralization defect since O.Th did not differ between groups. Because the animals were housed in same conditions of light cycle and received the same food *ad libitum*, it is likely that hypodynamia was directly responsible for hyper-osteoidosis although the mechanism remains unclear. Limitations of this study include lack of biochemical and molecular analyses (due to the absence of suitable tests or probes) and the absence of histodynamic bone labeling. In broiler chickens, walking has been reported to improve both the quality of meat and the quality of bones. Determination of ash weight (after calcination of long bones) and bone stiffness (measured by biomechanical tests) were increased, confirming that walking improves bone matrix quality [36, 45]. Another limitation of the study could be that avian bone differs from human bone at the microscopical level. In fact, several authors have reported similarities between avian and mammalian bones. Endochondral ossification of long bones in the tibia turkey has the same patterns of development underlying limb development in vertebrates [46]. It has been reported that bone fragility due to osteoporotic changes in laying hens represents an interesting model for human osteoporosis [47]. The Japanese quail, made hypodynamic by tight jackets, exhibit an altered bone remodeling with changes similar to other vertebrates including humans [48, 49]. A large amount of work has been done by the Lanyon’s group on the effects of disuse and compressive loads in the turkey [50, 51]. In fact, the main difference between avian and mammalian bone occurs at the onset of sexual maturity with the appearance of the medullary bone which acts as a reservoir of calcium for egg-laying birds [52]. In a hypodynamic model developed in 2-year-old laying hens, prolonged exercise restriction resulted in major bone loss together with a reduced biomechanical properties of long bones [53]. An increased resorption of medullary bone was responsible for reduced cortical thickness; mineral density was reported to be unchanged but this parameter was not determined by DXA. In the present study, broiler chickens were used and they do not possess medullary bone. As such, our observations are transposable to mammalian (and human bones).

New studies are needed with Raman and Fourier’s transformed infrared microspectroscopies to better characterize the alterations of the mineral and organic phase of the bone matrix. Also, the biomechanical properties of the bone material need to be fully characterized by newly developed methods [54]. We plan to conduct such studies both at the matrix level (by nanoindentation) and at the organ level (e.g., by 3-point bending on whole bones) on a new series of animals because these methods are destructive for the samples.

The confined chicken represents a new animal model of hypodynamia that can be used to study its effects on bone quality. The development of countermeasures, such as the return to free walking, may be beneficial in evaluating its effects on bone mass and bone quality.
